# The relationship between college students' belief in a just world and online prosocial behavior

**DOI:** 10.1002/pchj.788

**Published:** 2024-10-01

**Authors:** Yue Yang, Jun Zhan, Shanfang Liao, Rong Lian, Yiting Fang

**Affiliations:** ^1^ School of Psychology Zhejiang Normal University Jinhua PR China; ^2^ Zhejiang Philosophy and Social Science Laboratory for the Mental Health and Crisis Intervention of Children and Adolescents Zhejiang Normal University Jinhua PR China; ^3^ Key Laboratory of Intelligent Education Technology and Application of Zhejiang Province Zhejiang Normal University Jinhua PR China; ^4^ Postdoctoral Station of Psychology, School of Psychology Fujian Normal University Fuzhou PR China; ^5^ Fujian Agriculture and Forestry University Fuzhou PR China

**Keywords:** belief in a just world, college students, online prosocial behavior, sense of fairness, sense of unfairness

## Abstract

Previous studies have highlighted the critical role that the belief in a just world (BJW) plays in maintaining and promoting prosocial behaviors within individuals. Considered a stable personality trait, the crux of BJW lies in the conviction that individuals receive what they deserve, and deserve what they receive. Simultaneously, the relationship between BJW and prosocial behavior is impacted by an individual's sense of fairness or unfairness. However, past research has primarily focused on real‐life prosocial behavior, with limited exploration into the relationship between BJW and online prosocial behavior. This study, comprising a survey and an experiment, aimed to delve deeper into this relationship. The survey section randomly selected 4212 college students to examine how BJW correlates with online prosocial behavior. Findings predominantly revealed a significant positive correlation between online prosocial behavior and BJW. Additionally, the study explored how gender and place of origin influence these behaviors. Results showed that male students and those from urban areas exhibited significantly higher online prosocial behavior. The experimental research investigated the performance differences in online prosocial behaviors among college students under different fairness scenarios, revealing that the online prosocial behavior in an unfair situation was significantly higher than in fair or neutral situations. Furthermore, in unfair situations, a significant correlation was observed between BJW and online prosocial behavior. The findings from this study significantly advance our understanding of the dynamics between BJW and online prosocial behavior among college students, emphasizing that perceived injustices can markedly enhance prosocial behaviors in virtual settings. This study underscores the profound impact of fairness perceptions and highlights the modulating effects of gender and geographical background on online interactions.

## INTRODUCTION

The belief in a just world (BJW) is regarded as a stable personality trait, at the heart of which lies the individual's conviction that people get what they deserve, and what they get is what they should get, i.e., possessing a belief in a just world (Lerner & Miller, 1978). Past research has demonstrated that this belief contributes to the maintenance of social order and motivates individuals to engage in prosocial behavior (Bègue & Bastounis, 2003; Hafer & Bègue, 2005; Li et al., 2022; Zhang et al., 2023). Bègue and Bastounis (2003) found a positive correlation between the belief in a just world and prosocial behavior, suggesting that individuals who believe in a just world are more likely to engage in prosocial actions. They proposed that this might be because the belief in a just world can reduce individuals' fear of being exploited by others, thereby encouraging them to offer help to others more actively. Li et al. (2022) discovered that an individual's belief in a just world (BJW) moderates the relationship between social self‐efficacy and prosocial behavior, i.e., the positive link between social self‐efficacy and prosocial behavior is stronger when individuals have a strong belief in a just world. Individuals with a stronger belief in a just world are more inclined to solve problems with positive thoughts and attitudes, and they exhibit more prosocial behavior than others (Kaliuzhna, 2020). However, alternative viewpoints have also been proposed. Correia et al. (2007) found that when individuals feel that they are treated unfairly, their belief in a just world might be challenged, thereby reducing their prosocial behavior. In such cases, individuals might feel that their efforts do not receive just rewards, thus diminishing their prosocial behavior. Therefore, the relationship between the belief in a just world and prosocial behavior is not immutable.

Indeed, the sense of fairness and unfairness play a crucial role in the motivation for prosocial behavior. The sense of fairness, i.e., individuals' feeling that they are treated equitably and that the outcomes are commensurate with their contributions, can stimulate prosocial behavior. Numerous studies have revealed the positive impact of a sense of fairness on prosocial behavior (De Cremer & Van Lange, 2001; Gu et al., 2021; Tyler & Blader, 2003). For instance, Tyler and Blader (2003) found that a sense of fairness not only increases employee satisfaction but also enhances their work efficiency and teamwork, which are significant manifestations of prosocial behavior. De Cremer and Van Lange (2001) also confirmed that a sense of fairness can enhance people's loyalty and commitment to the group, further promoting prosocial behavior. Other research has verified that a sense of fairness significantly positively influences residents' prosocial behavior within the community (Gu et al., 2021). The impact of a sense of unfairness on prosocial behavior is the opposite. When individuals perceive unfairness, they may feel disappointed or frustrated with the existing social order, thereby reducing their prosocial behavior (Mulder et al., 2006). A sense of unfairness may lead individuals to believe that their efforts cannot receive just rewards, thereby reducing their help and support for others. However, this relationship is not always negative. For example, Hafer and Bègue (2005) proposed that when people face unfair situations, they might take action to rectify the unfairness and re‐establish the justice of the world (Hafer & Bègue, 2005). In other words, a sense of unfairness can sometimes act as a catalyst for stimulating prosocial behavior. These research findings suggest that the sense of fairness, sense of unfairness, and belief in a just world may collectively influence an individual's prosocial behavior.

Although past research has made some progress in exploring the relationship between the belief in a just world and real‐life prosocial behavior, a significant portion of these studies focus on prosocial behavior in real life, with relatively less discussion on online prosocial behavior. Examining prosocial behavior in online settings is increasingly critical as digital platforms become integral to daily communication and interaction. The unique characteristics of online environments, such as anonymity and the absence of physical cues, can significantly alter social behaviors and interactions (Valkenburg & Peter, 2009). Moreover, the internet serves as a vital space for the socialization of young people, shaping their behaviors and social norms in profound ways (Best et al., 2014). Understanding these behaviors is essential for addressing challenges like cyberbullying and social isolation. Furthermore, studying how perceptions of fairness influence online interactions can shed light on the dynamics of moral behavior in digital settings (Smith, 2012). Online prosocial behavior refers to positive actions such as support, help, and cooperation exhibited in an online environment. The Internet provides a relatively anonymous and free environment but also brings risks of virtualization and dispersion. People may display different behavior patterns in this environment compared to real life.

The goal of this study is to delve into the influence of the belief in a just world on college students' online prosocial behavior, and how the sense of fairness and unfairness modulates this influence. Specifically, we are interested in how the influence of the belief in a just world on online prosocial behavior differs when a sense of fairness and unfairness is induced. This study adopted two research methods to achieve its objectives. Firstly, a nationwide questionnaire survey was conducted, with approximately 4000 college students randomly selected as the subjects. This study aimed to reveal the overall situation of Chinese college students implementing online prosocial behavior and how the belief in a just world is associated with online prosocial behavior. This questionnaire survey sought to clarify the association pattern between the belief in a just world and online prosocial behavior through large‐scale sample data. Based on the results of the questionnaire survey, an online experiment was conducted as the second research method. This experiment adopted a single‐factor (sense of fairness vs. sense of unfairness vs. neutral induction) between‐subjects design, aiming to explore the performance differences in online prosocial behavior among college students under different fairness scenarios. It is worth noting that this experiment was conducted during the COVID‐19 lockdown, so all operations were completed online. By simulating fair and unfair situations, the experiment aimed to explore in depth how the sense of fairness and unfairness modulates the relationship between the belief in a just world and online prosocial behavior.

Moreover, the study examines the influence of gender and geographical background on online prosocial behavior. Research has identified significant gender differences in the use of digital technology and online interactions, which often reflect broader societal norms and expectations (Espinosa & Kovářík, 2015; Misbahudholam & Hardiansyah, 2022). Similarly, geographical differences, particularly between urban and rural areas, can significantly influence digital access and engagement, affecting how individuals participate in online communities (Wang & Si, 2024). These factors are crucial for understanding variations in online prosocial behavior, given their impact on digital literacy and access to technology.

## STUDY 1

### Methods

#### Participants

This study conducted a random sampling of college students nationwide through a questionnaire survey among the 4212 participants. To ensure the quality of the data, data cleaning and verification were carried out, excluding questionnaires that clearly violated normal answering habits, including missing answers, uniform answers, and excessively short answering time. In the end, 4082 valid questionnaires of college students (1866 males and 2216 females; 1172 from rural areas and 2970 from urban areas; age: 18 to 22 years, *M* = 19.961, *SD* = 1.507) were collected, serving as the basis for subsequent analysis.

#### Measures

##### Belief in a Just World Scale

This study plans to use the Belief in a Just World Scale compiled by Dalbert in 1999. The Chinese version was translated and revised by Su et al. (2012) and has been widely used in the Chinese population. This scale is mainly used to measure the degree of belief in a just world, with a total of 13 items, divided into two dimensions: general belief in a just world and personal belief in a just world. It uses a 6‐point Likert scoring method, with scores from 1 to 6 representing different degrees from “*strongly disagree*” to “*strongly agree*”. All items are scored positively, and the higher the total score and the scores of each dimension of the belief in a just world, the stronger the college students' belief in a just world. The internal consistency reliability of the total scale is indicated by a Cronbach's alpha of 0.885, with subscales for general and personal belief in a just world showing Cronbach's alpha coefficients of 0.794 and 0.845, respectively. The test–retest reliability of the total scale is 0.884, while the test–retest reliability coefficients for the general and personal belief in a just world subscales are 0.860 and 0.736, respectively. The revised scale demonstrates good reliability and validity (Su et al., 2012).

##### Online Prosocial Behavior Scale

This study uses the College Students' Online Altruistic Behavior Scale compiled by Zheng (2010). This scale has a total of 26 questions, including four dimensions: online support (9 items, such as “listening to netizens talk about their unhappiness and giving them guidance”), online guidance (6 items, such as “answering and guiding questions on the Internet”), online sharing (6 items, such as “posting timely and useful daily information online”), and online reminders (5 items, such as “reporting bad information online”). The scale uses a 4‐point scoring system, with scores from 1 to 4 representing *never*, *occasionally*, *usually*, and *always*, respectively. The higher the individual's score, the more online altruistic behavior they exhibit. The scale demonstrates high reliability and validity, with a Cronbach's alpha of 0.937 for the total scale, and the alpha coefficients for each dimension ranging from 0.800 to 0.878. Additionally, the test–retest reliability is 0.86, with the reliability of each dimension ranging from 0.71 to 0.81 (Zheng, 2010).

### Results

#### Difference analysis

In this study, there were significant differences in online prosocial behavior and belief in a just world among college students of different genders and places of origin (see Table 1).

**TABLE 1:** Difference analysis between variables.

	Gender				Place of origin			
	Male (*n* = 1866)	Female (*n* = 2216)	df	*t*	Urban (*n* = 1172)	Rural (*n* = 2910)	df	*t*
	*M* (*SD*)				*M* (*SD*)			
Online prosocial behavior	48.753 (15.300)	46.167 (12.803)	4080	5.878***	48.129 (14.481)	47.035 (13.873)	4080	2.249*
Online guidance	17.497 (3.797)	17.066 (3.146)	4080	8.825***	10.4292 (3.567)	10.292 (3.460)	4080	1.135
Online support	10.852 (5.653)	9.893 (5.057)	4080	2.572***	17.604 (5.587)	17.126 (5.234)	4080	2.59*
Online reminders	9.969 (3.436)	9.969 (3.021)	4080	7.138***	9.690 (3.309)	9.698 (3.309)	4080	1.513
Belief in a just world	52.707 (14.139)	54.018 (12.042)	4080	−3.199**	53.99 2 (13.071)	53.187 (13.046)	4080	1.783
General belief in a just world	23.859 (7.066)	7.066 (6.006)	4080	−4.451***	24.520 (6.606)	24.287 (6.495)	4080	1.032
Personal belief	28.848 (7.658)	7.658 (6.654)	4080	−1.785	29.473 (7.124)	28.900 (7.131)	4080	2.319*

*** Represents a significant correlation at level .001 (double‐tailed).

** Represents a significant correlation at level .01 (double‐tailed).

* Represents a significant correlation at level .05 (double‐tailed).

In terms of the level of online prosocial behavior, male students scored significantly higher than female students in the total score of online prosocial behavior and its various dimensions [total score of online prosocial behavior: *t*_(4080)_ = 5.878, *p < *.001, *d* = 0.18; online guidance: *t*_(4080)_ = 8.825, *p < *.001, *d* = 0.28; online support: *t*_(4080)_ = 2.572, *p < *.05, *d* = 0.08; online reminders: *t*_(4080)_ = 7.138, *p < *.001, *d* = 0.22]. In terms of place of origin, urban college students scored significantly higher than rural college students in online support, online sharing, and the total score of online prosocial behavior [total score of online prosocial behavior: *t*_(4080)_ = 2.249, *p < *.05, *d* = 0.07; online support: *t*_(4080)_ = 2.590, *p < *.05, *d* = 0.08; online sharing: *t*_(4080)_ = 2.824, *p < *.01, *d* = 0.28].

In terms of the level of belief in a just world, female students scored significantly higher than male students in general belief in a just world and the total score of belief in a just world [general belief in a just world: *t*_(4080)_ = 4.451 *p < *.001, *d* = 0.14; total score of belief in a just world: *t*_(4080)_ = 3.199, *p < *.01, *d* = 0.10]; rural college students scored significantly lower than urban college students in personal belief in a just world [*t*_(4080)_ = 3.199, *p < *.05, *d* = 0.03].

#### Correlation analysis

**FIGURE 1:**
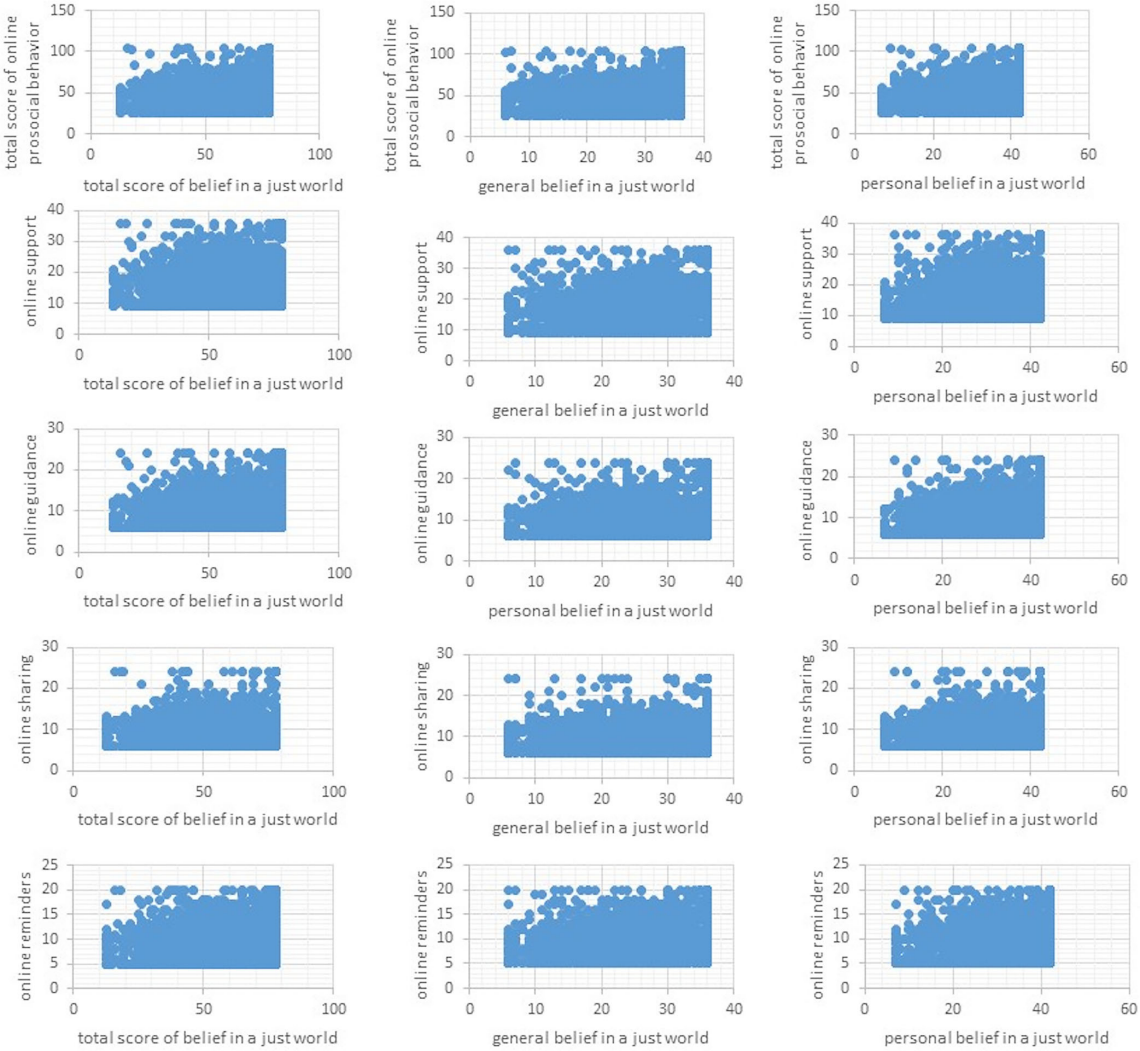
Scatter plot of correlation analysis between variables.

In this study, a significant positive correlation was found between college students' online prosocial behavior and their belief in a just world (see Table 2 Figure 1). This positive correlation was also significant in the analysis of each dimension (*p*s < .01).

**TABLE 2:** Correlation analysis matrix between variables.

Variable	Online support	Online guidance	Online sharing	Online reminders	Total score of online prosocial behavior
General belief in a just world	0.141**	0.120**	0.086**	0.155**	0.139**
Personal belief in a just world	0.133**	0.110**	0.085**	0.137**	0.128**
Total score of belief in a just world	0.143**	0.120**	0.089**	0.152**	0.139**

** Represents a significant correlation at level .01 (double‐tailed).

### Summary of study 1

This survey research discussed the relationships among gender, place of origin, online prosocial behavior, and belief in a just world. The results showed that, first, male college students' online prosocial behavior was significantly higher than that of females, and urban college students were also significantly more active in online behavior than rural college students. Second, in terms of belief in a just world, female college students scored higher than males, and urban college students scored higher than rural college students. In addition, we found a significant positive correlation between online prosocial behavior and belief in a just world, that is, the more college students believe in a just world, the more active they are in prosocial behavior in the online environment.

## STUDY 2

### Methods

#### Participants

This study recruited 105 undergraduate students to participate in the online experiment. Of these, 13 invalid data were eliminated, and a total of 92 valid participants were retained. In the study, the participants were divided into three groups based on the experimental conditions: the justice group, the neutral group, and the injustice group. The demographic breakdown for each group is as follows. Fairness Group: 15 males and 16 females, with 16 participants from urban areas and 15 from rural areas; Neutral Group: 12 males and 18 females, with 8 participants from urban areas and 22 from rural areas; Unfairness Group: 17 males and 14 females, with 14 participants from urban areas and 17 from rural areas.

#### Measures

##### Measurement tools for control variables

In addition to the belief in a just world, both material conditions and psychological capital emerge as potential determinants of an individual's perceived fairness (Newman et al., 2014; Tan et al., 2020). Typically, individuals with superior material resources tend to manifest elevated fairness perceptions, primarily attributable to the satisfaction of their basic life necessities, which in turn facilitates a more equitable viewpoint toward societal distribution. Furthermore, individuals with substantial psychological capital often exhibit a pronounced sense of fairness. This is attributed to their enhanced capability to navigate life's adversities and pressures, and a heightened conviction in their entitlement to equitable treatment (Furnham, 2003; Luthans et al., 2015; Semyonov et al., 2006). This study pre‐tested the above variables of the participants to eliminate the interference of group differences in irrelevant variables on the experimental induction effect. First, to obtain the variable information of material living standards, a self‐compiled material living standard survey form was designed and applied according to the objectives and specific needs of this study. This survey form mainly uses the related concept of family economic capital as the basis for measuring material living standards, covering economic information including individual living expenses, sources of living expenses, average monthly income per family member, family economic conditions, and main sources of family economic income, a total of 5 items. Secondly, the participants' psychological capital was evaluated. This study used the Positive Psychological Capital Questionnaire compiled by Zhang et al. (2010), which has 26 items and includes four dimensions: self‐efficacy, resilience, hope, and optimism. The questionnaire uses a 7‐point scale for scoring, with items 8, 10, 12, 14, and 25 scored in reverse. The higher the score from the questionnaire, the richer the positive psychological capital of the individual. The Cronbach's alpha coefficient of this questionnaire is 0.90, indicating good internal consistency. Finally, the Belief in a Just World Scale used in the above survey research was used to assess the level of belief in a just world of the participants.

##### Induction materials for the sense of justice or injustice

Before the formal experiment, a total of five news reports on justice, neutrality, and injustice were collected and sorted, and 20 college students were randomly selected to score their feelings after reading the materials from *very unjust* (1 point) to *very just* (7 points), the higher the score, the stronger the experience of justice. A single‐factor repeated measures analysis of variance showed that there were significant differences in the scores of the participants for the three types of materials [*F*_(2,63)_ = 126.398, *p < *.001, *η* ^*2*^ = 0.927], and post‐hoc comparisons confirmed that the scores for the justice materials were significantly higher than those for the injustice materials and neutral materials (*p < *.001), and the scores for the neutral materials were significantly higher than those for the injustice materials (*p < *.001).

##### Online prosocial behavior intention task

The “Helping Forward” paradigm was used to evaluate the participants' tendency towards online prosocial behavior (Fan, 2020; Yue, 2015). After the participants browsed job‐seeking resources, online fraud help, patient help, and online donation information, they needed to score their willingness to forward each online help information, from 1 (*very unwilling*) to 7 (*very willing*). The average willingness to help provided an evaluation of online prosocial behavior intention; the higher the score, the stronger the online prosocial behavior intention of the participants.

#### Experimental design and procedures

Due to the impact of the COVID‐19 epidemic in November 2022, this study was conducted in the form of an online experiment, and all tasks were completed one‐on‐one through WeChat video and the Questionnaire Star platform (wjx.com). Participants were required to read and fill out the informed consent form before the experiment began. During the WeChat video sessions, each participant was individually connected to an experimenter who monitored the session in real time to ensure compliance and engagement. This setup allowed for immediate interaction and feedback, simulating a controlled yet naturalistic setting that enhanced the reliability of the induced emotional states. Special attention was given to ensuring that all participants were adequately informed about how to manage the technical aspects of the video call, such as camera positioning and audio settings, to maintain a high quality of communication throughout the experiment. First, a pre‐test was conducted on psychological capital, material living standards, and belief in a just world. Then the participants were randomly assigned to one of three groups, with 31 people in each of the justice group and the injustice group, and 30 people in the neutral group, and the participants were asked to read materials that induced a sense of justice, injustice, or neutrality. Finally, to ensure the effectiveness of the measurement of online prosocial behavior intention, an unpaid online help task that seemed unrelated to this experiment was set up. After filling out the above questionnaires, the participants will see the prompt “This experiment has ended, thank you for your cooperation”. Then there are four online help information resources at the bottom of the same page, and the participants need to read and fill in the degree of willingness to help.

### Results

#### Experimental manipulation examination

Initially, variance analyses were conducted with psychological capital, material living standards, and belief in a just world as dependent variables, and group (fairness group vs. unfairness group vs. neutral group) as an independent variable. The results revealed no significant intergroup differences for the aforementioned dependent variables (psychological capital: *F*_(2,89)_ = 0.599, *p > *.05, *η* ^*2*^ = 0.013; material living standards: *F*_(2,89)_ = 2.036, *p > *.05, *η* ^*2*^ = 0.044; belief in a just world: *F*_(2,89)_ = 0.861, *p > *.05, *η* ^*2*^ = 0.019), thereby excluding the potential interference of related extraneous variables on the experimental effects.

Subsequently, an analysis of variance was conducted with the induced sense of fairness from the material as a dependent variable and group (fairness group vs. unfairness group vs. neutral group) as an independent variable. Significant intergroup differences were discovered [*F*_(2,89)_ = 9.501, *p < *.001, *η* ^*2*^ = 0.176], with post‐hoc comparison indicating that the sense of fairness in the fairness group was significantly higher than in the unfairness and neutral groups (*p*s < .001), and was significantly greater in the neutral group than in the unfairness group (*p < *.001).

#### Difference analysis

**FIGURE 2:**
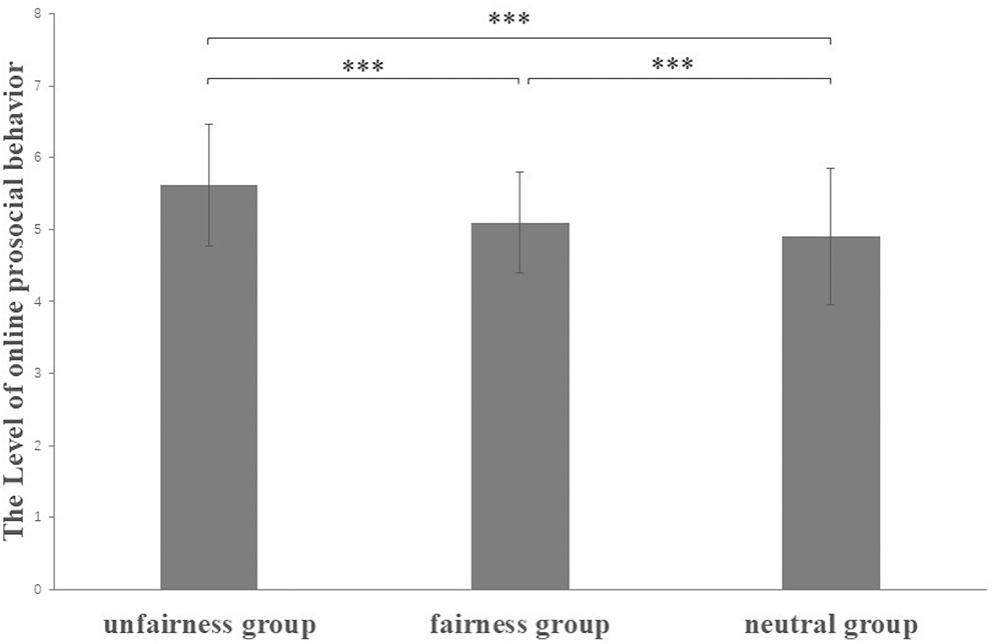
Comparisons of online prosocial behavior among three conditions. Error bars represent (−1)/(+ 1) SE; *** *p* < .001.

Analysis of variance was conducted with the group (induced sense of fairness vs. induced unfairness vs. induced neutral emotion) as an independent variable and online prosocial behavior intention as a dependent variable. Significant intergroup differences were found [*F*_(2,89)_ = 451.763, *p < *.001, *η* ^*2*^ = 0.910]. Post‐hoc comparison showed that the online prosocial behavior intention of the unfairness group was significantly higher than the neutral group and fairness group (*p*s < .01) (see Figure 2). This suggests that, compared to the sense of fairness and neutral emotions, the sense of unfairness is more likely to evoke online prosocial behavior in college students.

#### Correlation analysis

**FIGURE 3:**
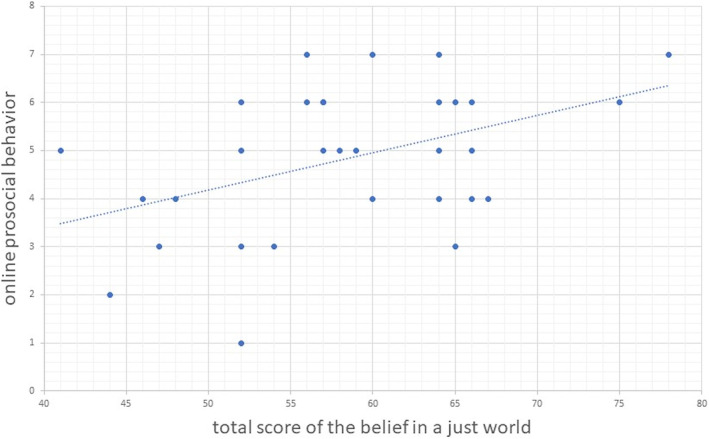
Scatter plot of correlation analysis between online prosocial behavior and the total score of the belief in a just world of the unfairness group.

Correlation analyses were separately performed on online prosocial behavior and the belief in a just world for the fairness group, unfairness group, and neutral group. The results showed that there was a significant correlation between college students' online prosocial behavior and the total score of the belief in a just world of the unfairness group (*r* = 0.438, *p < *.05; see Figure 3), marginal significant correlations were found with both general belief in a just world and personal belief in a just world (*r* = 0.336, *p = *.064; *r* = 0.307, *p = *.093). However, no such correlations were detected in the fairness group (total score of the belief in a just world: *r* = 0.169, *p = *.364; general belief: *r* = 0.156, *p = *.401; personal belief: *r* = 0.156, *p = *.402) and the neutral group (total score of the belief in a just world: *r* = 0.228, *p = *.225; general belief: *r* = −0.282, *p = *.131; personal belief: *r* = −0.163, *p = *.391).

### Summary of study 2

This study revealed the impact of the senses of justice and injustice on online prosocial behavior and explored the relationship between the belief in a just world and online prosocial behavior in unjust situations. The findings suggest that a sense of injustice can significantly stimulate online prosocial behavior, while a sense of justice can also promote online prosocial behavior to some extent. Moreover, in unjust situations, the relationship between the belief in a just world and online prosocial behavior becomes more significant.

## GENERAL DISCUSSION

### Impact of gender and place of origin on online prosocial behavior and belief in a just world

This study found that male college students demonstrated significantly higher online prosocial behavior than female college students. This is consistent with the research results of Zheng (2010) and also supports the view of social role theory, which posits that due to socialization processes, men are more likely to exhibit traits of independence, autonomy, and leadership (Eagly, 2013). These traits may drive them to be more willing to share and guide others in an online environment. On the other hand, urban‐born college students scored significantly higher in terms of online support, online sharing, and total online prosocial behavior than their rural counterparts. This may be related to the more frequent use of the internet and richer online resources among urban students (Valenzuela et al., 2009; Wang et al., 2021). This supports the digital divide theory, which suggests a significant unequal distribution of internet usage and skills among groups with different socio‐economic status (Norris, 2003).

Furthermore, female college students showed significantly higher scores in the general belief in a just world and the total score of the belief in a just world than male students. This aligns with the research of Dalbert (1999) and also concurs with the gender difference theory proposed by sociological research, which suggests that due to socialization and physiological differences, women tend to pay more attention to fairness, justice, and morality (Dalbert, 1999; Gilligan, 1982). Additionally, rural college students scored significantly lower in personal belief in a just world compared to urban students. The reason may lie in the fact that rural college students have experienced relatively more life hardships and social injustices, thus forming a cognitive attitude adapted to these circumstances. This phenomenon might be explained from the perspective of compensatory control theory, which posits that when individuals' worldview of justice is threatened, they adjust their beliefs to adapt to an unjust reality. These compensatory control processes help people cope with the anxiety and unease caused by the lack of personal control (Kay et al., 2005; Zhou & Guo, 2013).

### The relationship between online prosocial behavior and belief in a just world

Firstly, the survey part of this study found a significant positive correlation between college students' online prosocial behavior and their belief in a just world. This means that students who believe in a just world are more likely to exhibit altruistic behavior in an online environment. This finding is in line with the just world theory and social exchange theory (Emerson, 1976; Lerner, 1980). The just world theory posits that people who believe the world is just are more likely to engage in prosocial behavior because they believe their good deeds will be rewarded. Social exchange theory, on the other hand, suggests that people expect some form of return when engaging in prosocial behavior. This positive correlation could also be associated with the self‐determination theory (Ryan & Deci, 2000), which emphasizes that intrinsic motivation is a significant driving force behind prosocial behavior. That is, when people engage in prosocial behavior due to intrinsic values and moral beliefs (such as belief in a just world), their behavior may be more enduring and intense. This may explain why students who believe in a just world are more likely to engage in prosocial behavior online. Furthermore, this could be related to the unique characteristics of online environments, such as anonymity and extensive social connections. In online environments, people can express their opinions and behaviors more freely without worrying about social evaluation and pressure (Bargh et al., 2002). This may make the impact of the belief in a just world more evident in online environments, especially for those students who strongly believe in the justice of the world. Simultaneously, the internet offers extensive social connections, allowing prosocial behavior to influence more people, thereby extending the impact of belief in a just world. Finally, this positive correlation may also be a bidirectional process. That is, online prosocial behavior is not only a result of the belief in a just world but may also, in turn, reinforce college students' belief in a just world. When college students see their prosocial behavior receiving positive feedback and rewards, they may believe more in the fairness of the world, further promoting more online prosocial behavior.

Secondly, the experimental part of this study confirmed, on the one hand, that feelings of justice, injustice, and neutral emotions have differential impacts on online prosocial behavior. On the other hand, it reconfirmed the significant positive correlation between belief in a just world and online prosocial behavior. However, this correlation is more pronounced when feelings of injustice are induced. The group that was made to feel unjust had a significantly higher performance in online prosocial behavior than other groups. This observation reveals the stimulating effect of a sense of injustice on the online prosocial behavior of college students, which is consistent with many past studies (Guo et al., 2022; Hafer & Bègue, 2005; Martinson et al., 2006; Simon & Klandermans, 2001; Tangney et al., 2007).

Motivation theory argues that human behavior is driven by internal needs and desires (Ryan & Deci, 2000). When individuals face an unjust situation, they may experience a sense of imbalance, which might motivate them to take action to restore balance. This balance could be achieved by engaging in prosocial behavior, as this behavior can help individuals construct a positive self‐image and alleviate their dissatisfaction with the unjust situation. Furthermore, this tendency to participate in prosocial behavior could also be interpreted as a manifestation of social responsibility. When faced with injustice, individuals might feel a sense of social responsibility that drives them to act to change the situation (Bury, 1982; Kahneman et al., 1991; Marmot et al., 2008; Mathur et al., 2022; Rossa‐Roccor et al., 2021). In online environments, this behavior might manifest as sharing beneficial information, participating in public welfare activities, and other prosocial behaviors. Therefore, when confronted with injustices online, individuals might be more inclined to engage in prosocial behavior, hoping to improve the situation through their efforts. Moreover, according to the just world theory, people tend to believe that the world is just, and the face of injustice challenges this belief (Lerner, 1980). Thus, when facing injustice, people might be more active in engaging in prosocial behavior to uphold their belief in a just world. In the face of injustice, people might strive to restore justice in various ways, including prosocial behaviors (like donating or volunteering), and possibly even self‐sacrifice (Furnham, 2003; Hafer & Bègue, 2005; Montada & Lerner, 1998). It is worth noting that while a sense of injustice can enhance online prosocial behavior, this does not mean that we should deliberately create unjust situations to enhance prosocial behavior. On the contrary, we should strive to create a fair online environment and encourage just and friendly behavior.

Additionally, while prosocial behavior induced by a sense of justice did not reach the level of those induced by a sense of injustice, it was still higher than that induced by neutral emotions. This result suggests that a sense of justice also has a certain positive effect, possibly motivating college students to participate in online prosocial behavior to a certain extent. This might be related to the satisfaction and positive emotions generated by a sense of justice, potentially guiding individuals to choose actions more beneficial to others and the community (De Cremer & Van Lange, 2001; Wenzel et al., 2012). Besides, a sense of justice fulfills people's need for what they feel is “deserved,” which could guide them to engage in more prosocial behavior. A fair environment might also potentially reinforce the belief in a just world, making individuals more willing to engage in prosocial behavior (Gollwitzer et al., 2009; Lerner, 1980; Mikula et al., 1998; Wenzel & Thielmann, 2006).

Another important finding of this study is that the relationship between belief in a just world and online prosocial behavior becomes more significant when a sense of injustice is induced. This might be because when people feel unjust, their belief in a just world could stimulate their fair behavior (Bègue & Bastounis, 2003; Hafer & Bègue, 2005). The belief in a just world is a broad psychological construct that affects individuals' understanding of themselves, others, and the world. When individuals believe the world is just, they tend to think people usually get what they deserve (Lerner, 1980). When faced with injustice, their belief in a just world might be challenged. This belief might encourage them to restore justice through actions, thus re‐establishing the world's fairness. Compared to a fair environment, an unfair one might more intensively stimulate an individual's belief in a just world, thereby driving them more strongly to engage in prosocial behavior. In a fair environment, as there is no need to restore any injustice, the belief in a just world might not stimulate prosocial behavior as intensively. This could explain why in unjust situations, the belief in a just world seems more critical in promoting online prosocial behavior.

## LIMITATIONS

This study has combined questionnaire surveys and experimental research to comprehensively explore the relationship between the belief in a just world and prosocial behavior online, yielding a series of enlightening conclusions. However, certain limitations in the design and implementation of this study are inevitably present.

Firstly, this research involves only a random sampling of Chinese college students, without suitable matching and control for potential influencing factors such as place of origin and type of institution. This somewhat limits the generalizability and applicability of the research results. Future research should pay more attention to the diversity of samples in order to enhance the universality and accuracy of research results.

Secondly, the second part of this study adopts an online experimental research paradigm. Compared to laboratory research, this paradigm presents certain challenges in observing and controlling the participation status of subjects. Therefore, we suggest that subsequent research attempts to use laboratory research paradigms to validate and extend the findings of this study.

Lastly, it is worth noting that, although this research highlights the positive role of belief in a just world in promoting prosocial behavior online, some prior studies (e.g., Furnham, 2003) have pointed out that over‐reliance on the belief in a just world could lead to hypersensitivity to injustice, resulting in unnecessary anxiety and stress (Furnham, 2003). This aspect has not been fully explored and analyzed in this study. Hence, future research needs to further scrutinize the potential negative impacts that the belief in a just world might bring, so that we can more comprehensively understand the complex relationship between the belief in a just world and prosocial behavior online.

## CONCLUSION

This study specifically investigates the inherent relationship between prosocial behavior online and the belief in a just world. Our findings indicate that male and urban‐origin college students exhibit higher levels of prosocial behavior online, while females and city‐born students are more likely to strongly uphold the belief that the world is fair. Significantly, a notable positive correlation between online prosocial behavior and belief in a just world was observed. Feelings of justice or injustice induced varied impacts on prosocial behavior online. When individuals perceived injustice, the connection between their belief in a just world and online prosocial behavior appeared to be more pronounced. These important findings unravel the influence of gender, place of origin, and belief in a just world on prosocial behavior online, offering valuable insights for the design of more effective online behavioral norms and educational strategies. However, to confirm these discoveries, we need to conduct further research and delve deeper into other potential factors affecting online prosocial behavior and belief in a just world.

## CONFLICT OF INTEREST STATEMENT

The authors declare that the research was conducted in the absence of any commercial or financial relationships that might be construed as a conflict of interest.

## ETHICS STATEMENT

The whole study was approved by the Institutional Review Board of the Department of Psychology, Fujian Normal University and Fujian Agriculture and Forestry University, China, and all participants signed informed consent forms.

## References

[pchj788-bib-0001] Bargh, J. A. , McKenna, K. Y. , & Fitzsimons, G. M. (2002). Can you see the real me? Activation and expression of the “true self” on the internet. Journal of Social Issues, 58(1), 33–48. 10.1111/1540-4560.00247

[pchj788-bib-0002] Bègue, L. , & Bastounis, M. (2003). Two spheres of belief in justice: Extensive support for the bidimensional model of belief in a just world. Journal of Personality, 71(3), 435–463. 10.1111/1467-6494.7103007 12762422

[pchj788-bib-0003] Best, P. , Manktelow, R. , & Taylor, B. (2014). Online communication, social media and adolescent wellbeing: A systematic narrative review. Children and Youth Services Review, 41, 27–36. 10.1016/j.childyouth.2014.03.001

[pchj788-bib-0004] Bury, M. (1982). Chronic illness as biographical disruption. Sociology of Health & Illness, 4(2), 167–182. 10.1111/1467-9566.ep11339939 10260456

[pchj788-bib-0005] Correia, I. , Vala, J. , & Aguiar, P. (2007). Victim's innocence, social categorization, and the threat to the belief in a just world. Journal of Experimental Social Psychology, 43(1), 31–38. 10.1016/j.jesp.2005.12.010

[pchj788-bib-0006] Dalbert, C. (1999). The world is more just for me than generally: About the personal belief in a just world scale's validity. Social Justice Research, 12, 79–98. 10.1007/978-1-4757-3383-9_3

[pchj788-bib-0007] De Cremer, D. , & Van Lange, P. A. (2001). Why prosocials exhibit greater cooperation than proselfs: The roles of social responsibility and reciprocity. European Journal of Personality, 15(1_suppl), S5–S18. 10.1002/per.418

[pchj788-bib-0008] Eagly, A. H. (2013). Sex differences in social behavior: Comparing social role theory and evolutionary psychology. American Psychologist, 52, 1380–1383. 10.1037/0003-066X.52.12.1380.b 9414607

[pchj788-bib-0009] Emerson, R. (1976). Social exchange theory. Annual Review of Sociology, 2(1), 335–362. 10.1146/annurev.so.02.080176.002003

[pchj788-bib-0010] Espinosa, M. P. , & Kovářík, J. (2015). Prosocial behavior and gender. Frontiers in Behavioral Neuroscience, 9, 88. 10.3389/fnbeh.2015.00088 25926783 PMC4396499

[pchj788-bib-0011] Fan, Y. (2020). Personal oriented values and online prosocial behavior: A moderated mediation model from the perspective of self‐determination. Master's thesis. East China Normal University. 10.12677/AP.2022.1211452

[pchj788-bib-0012] Furnham, A. (2003). Belief in a just world: Research progress over the past decade. Personality and Individual Differences, 34(5), 795–817. 10.1016/S0191-8869(02)00072-7

[pchj788-bib-0013] Gilligan, C. (1982). In a different voice. Harvard UniversityPress. 10.4159/9780674037618-003

[pchj788-bib-0014] Gollwitzer, M. , Rothmund, T. , Pfeiffer, A. , & Ensenbach, C. (2009). Why and when justice sensitivity leads to pro‐ and antisocial behavior. Journal of Research in Personality, 43(6), 999–1005. 10.1016/j.jrp.2009.07.003

[pchj788-bib-0015] Gu, X. , Chen, H. , & Huang, Y. (2021). The influence of perceived justice on prosocial behavior of community residents under recall priming paradigm. In Abstracts of the 23rd National Conference on psychology (part 1).

[pchj788-bib-0016] Guo, Y. , Chen, X. , Ma, J. , Li, Y. , & Hommey, C. (2022). How belief in a just world leads to prosocial behaviours: The role of communal orientation. Personality and Individual Differences, 195, 111642. 10.1016/j.paid.2022.111642

[pchj788-bib-0017] Hafer, C. L. , & Bègue, L. (2005). Experimental research on just‐world theory: Problems, developments, and future challenges. Psychological Bulletin, 131(1), 128–167. 10.1037/0033-2909.131.1.128 15631556

[pchj788-bib-0018] Kahneman, D. , Knetsch, J. L. , & Thaler, R. H. (1991). Anomalies: The endowment effect, loss aversion, and status quo bias. Journal of Economic Perspectives, 5(1), 193–206. 10.1257/jep.5.1.193

[pchj788-bib-0019] Kaliuzhna, M. (2020). Symmetries and asymmetries in the belief in a just world. Personality and Individual Differences, 161, 109940. 10.1016/j.paid.2020.109940

[pchj788-bib-0020] Kay, A. C. , Jost, J. T. , & Young, S. (2005). Victim derogation and victim enhancement as alternate routes to system justification. Psychological Science, 16(3), 240–246. 10.1111/j.0956-7976.2005.00810.x 15733206

[pchj788-bib-0022] Lerner, M. J. (1980). The belief in a just world. Springer. 10.1007/978-1-4899-0448-5_2

[pchj788-bib-0023] Lerner, M. J. , & Miller, D. T. (1978). Just world research and the attribution process: Looking back and ahead. Psychological Bulletin, 85(5), 1030–1051. 10.1037/0033-2909.85.5.1030

[pchj788-bib-0024] Li, L. , Liu, H. , Wang, G. , Chen, Y. , & Huang, L. (2022). The relationship between ego depletion and prosocial behavior of college students during the COVID‐19 pandemic: The role of social self‐efficacy and personal belief in a just world. Frontiers in Psychology, 13, 801006. 10.3389/fpsyg.2022.801006 35548506 PMC9083063

[pchj788-bib-0025] Luthans, F. , Youssef, C. M. , & Avolio, B. J. (2015). Psychological capital and beyond. Oxford University Press. 10.1093/acprof:oso/9780195187526.003.0001

[pchj788-bib-0026] Marmot, M. , Friel, S. , Bell, R. , Houweling, T. A. , & Taylor, S. (2008). Closing the gap in a generation: Health equity through action on the social determinants of health. The Lancet, 372(9650), 1661–1669. 10.1016/S0140-6736(08)61690-6 18994664

[pchj788-bib-0027] Martinson, B. C. , Anderson, M. S. , Crain, A. L. , & De Vries, R. (2006). Scientists' perceptions of organizational justice and self‐reported misbehaviors. Journal of Empirical Research on Human Research Ethics, 1(1), 51–66. 10.1525/jer.2006.1.1.51 PMC1483900 16810337

[pchj788-bib-0028] Mathur, V. A. , Trost, Z. , Ezenwa, M. O. , Sturgeon, J. A. , & Hood, A. M. (2022). Mechanisms of injustice: What we (do not) know about racialized disparities in pain. Pain, 163(6), 999–1005. 10.1097/j.pain.0000000000002528 34724680 PMC9056583

[pchj788-bib-0029] Mikula, G. , Scherer, K. R. , & Athenstaedt, U. (1998). The role of injustice in the elicitation of differential emotional reactions. Personality and Social Psychology Bulletin, 24(7), 769–783. 10.1177/0146167298247009

[pchj788-bib-0030] Misbahudholam, A. R. M. , & Hardiansyah, F. (2022). Prosocial behavior of elementary school students based on gender differences in society 5.0. Journal of Innovation in Educational and Cultural Research, 3(3), 390–396. 10.46843/jiecr.v3i3.121

[pchj788-bib-0031] Montada, L. , & Lerner, M. J. (1998). Responses to victimizations and belief in a just world. Springer Science & Business Media. 10.1007/978-1-4757-6418-5

[pchj788-bib-0032] Mulder, L. B. , Van Dijk, E. , De Cremer, D. , & Wilke, H. A. (2006). Undermining trust and cooperation: The paradox of sanctioning systems in social dilemmas. Journal of Experimental Social Psychology, 42(2), 147–162. 10.1016/j.jesp.2005.03.002

[pchj788-bib-0033] Newman, A. , Ucbasaran, D. , Zhu, F. , & Hirst, G. (2014). Psychological capital: A review and synthesis. Journal of Organizational Behavior, 35(S1), S120–S138. 10.1002/job.1916

[pchj788-bib-0034] Norris, P. (2003). Digital divide: Civic engagement, information poverty, and the internet worldwide. University of Toronto Press.

[pchj788-bib-0035] Rossa‐Roccor, V. , Giang, A. , & Kershaw, P. (2021). Framing climate change as a human health issue: Enough to tip the scale in climate policy? The Lancet Planetary Health, 5(8), e553–e559. 10.1016/S2542-5196(21)00113-3 34390673

[pchj788-bib-0036] Ryan, R. M. , & Deci, E. L. (2000). Self‐determination theory and the facilitation of intrinsic motivation, social development, and well‐being. American Psychologist, 55(1), 68–78. 10.1037/0003-066X.55.1.68 11392867

[pchj788-bib-0037] Semyonov, M. , Raijman, R. , & Gorodzeisky, A. (2006). The rise of anti‐foreigner sentiment in European societies, 1988–2000. American Sociological Review, 71(3), 426–449. 10.1177/000312240607100304

[pchj788-bib-0038] Simon, B. , & Klandermans, B. (2001). Politicized collective identity: A social psychological analysis. American Psychologist, 56(4), 319–331. 10.1037/0003-066X.56.4.319 11330229

[pchj788-bib-0039] Smith, P. K. (2012). Cyberbullying and cyber aggression. In S. Jimerson , A. Nickerson , M. J. Mayer , & M. J. Furlong (Eds.), Handbook of school violence and school safety (pp. 93–103). Routledge. 10.4324/9780203841372.ch8

[pchj788-bib-0040] Su, Z. , et al. (2012). Revision of the Belief in a Just World Scale and a study of its reliability and validity among college students. Chinese Journal of Behavioral Medicine and Brain Science, 21(6), 561–563. 10.3760/cma.j.issn.1674-6554.2012.06.026

[pchj788-bib-0041] Tan, J. J. , Kraus, M. W. , Carpenter, N. C. , & Adler, N. E. (2020). The association between objective and subjective socioeconomic status and subjective well‐being: A meta‐analytic review. Psychological Bulletin, 146(11), 970–1020. 10.1037/bul0000258 33090862

[pchj788-bib-0042] Tangney, J. P. , Stuewig, J. , & Mashek, D. J. (2007). Moral emotions and moral behavior. Annual Review of Psychology, 58, 345–372. 10.1146/annurev.psych.56.091103.070145 PMC3083636 16953797

[pchj788-bib-0043] Tyler, T. R. , & Blader, S. L. (2003). The group engagement model: Procedural justice, social identity, and cooperative behavior. Personality and Social Psychology Review, 7(4), 349–361. 10.1207/S15327957PSPR0704_07 14633471

[pchj788-bib-0044] Valenzuela, S. , Park, N. , & Kee, K. F. (2009). Is there social capital in a social network site?: Facebook use and college students' life satisfaction, trust, and participation. Journal of Computer‐Mediated Communication, 14(4), 875–901. 10.1111/j.1083-6101.2009.01474.x

[pchj788-bib-0045] Valkenburg, P. M. , & Peter, J. (2009). Social consequences of the internet for adolescents: A decade of research. Current Directions in Psychological Science, 18(1), 1–5. 10.1111/j.1467-8721.2009.01595.x

[pchj788-bib-0046] Wang, C. , & Si, L. (2024). The intersection of public policy and public access: Digital inclusion, digital literacy education, and libraries. Sustainability, 16(5), 1878. 10.3390/su16051878

[pchj788-bib-0047] Wang, S. , Fu, L. , & Shen, M. (2021). Performance, cause and implication of the second digital divide among contemporary college students. Documentation, Information and Knowledge, 38(1), 125–135. 10.14725/jenc.v3n1a1068

[pchj788-bib-0048] Wenzel, M. , Okimoto, T. G. , & Cameron, K. (2012). Do retributive and restorative justice processes address different symbolic concerns? Critical Criminology, 20, 25–44. 10.1007/s10612-011-9147-7

[pchj788-bib-0049] Wenzel, M. , & Thielmann, I. (2006). Why we punish in the name of justice: Just desert versus value restoration and the role of social identity. Social Justice Research, 19, 450–470. 10.1007/s11211-006-0028-2

[pchj788-bib-0051] Yue, Y. (2015). The effect of trait empathy and empathy on online altruistic behavior of college students. Master's Thesis (Vol. 50, pp. 1051–1060). Central China Normal University.

[pchj788-bib-0052] Zhang, K. , Zhang, S. , & Dong, Y. (2010). Positive psychological capital: Measurement and relationship with mental health. Psychological and Behavioral Research, 8(1), 58–64.

[pchj788-bib-0053] Zhang, W. , Chen, Y. , & Zhu, L. (2023). “Attraction of the like”: The influence of peer's donation choice on prosocial behavior of adolescents and the role of the belief in a just world. Acta Psychologica Sinica, 55(9), 1453–1464. 10.3724/SP.J.1041.2023.01453

[pchj788-bib-0054] Zheng, X. (2010). Online altruistic behavior of college students: Scale development and multilevel linear analysis. Doctoral dissertation. Shanghai Normal University. 10.7666/d.y1667767

[pchj788-bib-0055] Zhou, C. , & Guo, Y. (2013). Belief in a just world: A double‐edged sword for justice restoration. Advances in Psychological Science, 21(1), 144. 10.3724/SP.J.1042.2013.00144

